# The resilient intensive care unit

**DOI:** 10.1186/s13613-022-01011-x

**Published:** 2022-04-26

**Authors:** Jorge I. F. Salluh, Pedro Kurtz, Leonardo S. L. Bastos, Amanda Quintairos, Fernando G. Zampieri, Fernando A. Bozza

**Affiliations:** 1 D’Or Institute for Research and Education (IDOR), Rua Diniz Cordeiro, 30 – 3º andar, Rio de Janeiro, RJ Brazil; 2 Postgraduate Program of Internal Medicine, Federal University of Rio de Janeiro, (UFRJ), Rio de Janeiro, RJ Brazil; 3 Hospital Copa Star, Rio de Janeiro, RJ Brazil; 4 Paulo Niemeyer State Brain Institute (IECPN), Rio de Janeiro, RJ Brazil; 5 Department of Industrial Engineering (DEI), Pontifical Catholic University of Rio de Janeiro (PUC-Rio), Rio de Janeiro, RJ Brazil; 6 Department of Critical and Intensive Care Medicine, Academic Hospital Fundación Santa Fe de Bogota, Bogota, Colombia; 7 HCor Research Institute, Sao Paulo, Brazil; 8 National Institute of Infectious Disease Evandro Chagas (INI), Oswaldo Cruz Foundation (FIOCRUZ), Rio de Janeiro, RJ Brazil

## Abstract

**Background:**

The COVID-19 pandemic tested the capacity of intensive care units (ICU) to respond to a crisis and demonstrated their fragility. Unsurprisingly, higher than usual mortality rates, lengths of stay (LOS), and ICU-acquired complications occurred during the pandemic. However, worse outcomes were not universal nor constant across ICUs and significant variation in outcomes was reported, demonstrating that some ICUs could adequately manage the surge of COVID-19.

**Methods:**

In the present editorial, we discuss the concept of a resilient Intensive Care Unit, including which metrics can be used to address the capacity to respond, sustain results and incorporate new practices that lead to improvement.

**Results:**

We believe that a resiliency analysis adds a component of preparedness to the usual ICU performance evaluation and outcomes metrics to be used during the crisis and in regular times.

**Conclusions:**

The COVID-19 pandemic demonstrated the need for a resilient health system. Although this concept has been discussed for health systems, it was not tested in intensive care. Future studies should evaluate this concept to improve ICU organization for standard and pandemic times.

## Introduction

The COVID-19 pandemic tested the capacity of intensive care units (ICU) to respond to a crisis and demonstrated their fragility. An exceptionally high number of severely ill-patients overwhelmed hospitals and ICUs, and despite the increase of ICU beds, the access to critical care was not straightforward. Quantitative and qualitative deficits in staff, material resources, as well as a higher variation of standards of care delivery were reported [1, 2]. Unsurprisingly, higher than usual mortality rates, lengths of stay (LOS), and ICU-acquired complications occurred during the pandemic. However, worse outcomes were not universal nor constant across ICUs. Indeed, significant variation in outcomes was reported demonstrating that despite the challenges, and some ICUs could adequately manage the surge of COVID-19 [3].

In recent years, the resilience of health systems was tested multiple times, and yet, despite previous experiences with Influenza and Ebola, the COVID-19 pandemic showed that the main prerequisites for ICU resilience were usually not present. A resilient health system is defined by the capacity of its stakeholders and institutions to prepare, adapt and respond to a crisis [4]. This response should aim to sustain core operations, learn from the crisis, and produce good outcomes.

## What is a resilient ICU?

A resilient ICU must be adaptable and capable of responding not only to a major calamity such as a pandemic but also to more frequent struggles, such as changes in case-mix and increases in the volume of admissions. Therefore, it is reasonable to assume that a resilient ICU must have the ability to adapt to sudden changes of case mix, severity, and volume with minimal impact on clinical outcomes. In addition to adaptation, a resilient ICU must rapidly learn and implement measures to sustain good results over time. The incorporation of new practices learned during the crisis may drive improved performance despite the continuity of the situation. In a resilient environment, crisis-response should be coupled with better personnel management aiming at staff wellness. In the COVID-19 pandemic, an enormous psychological burden on healthcare workers [5, 6] occurred and could have been mitigated by reducing the pressure on the ICU through better management of resources [7, 8].

One general approach to defining health systems resilience is based on the 4S (staff, stuff, space, systems) [9]. Using the COVID-19 pandemic as an example, a resilient ICU would be the one that guaranteed the 4S (therefore being able to cope with a surge of critically ill-patients) and ensured that evidence-based practices, while incorporating the recently generated knowledge, such as corticosteroids and non-invasive ventilation [10] simultaneously refraining from prescribing non-evidence-based interventions (i.e., HCQ, ivermectin, etc.). Therefore, we believe that an additional “S” (for science) could be added to the “4S” as the generation of new evidence through research and its incorporation in practice via quality improvement projects are a fundamental part of the learning and improvement process of a resilient ICU. Hollnagel´s Resilience Assessment Grid (RAG) includes the “scientific (learning)” aspect when it defines resilience performance in 4 pillars: learn, monitor, anticipate, and respond [7].

## Assessing ICU resiliency: a proposed framework

What metrics can be used to address the capacity to “anticipate”, “respond” and “incorporate new practices that lead to improvement (learn)”? Albeit imperfect, some potential indicators can be proposed.

**Fig. 1:**
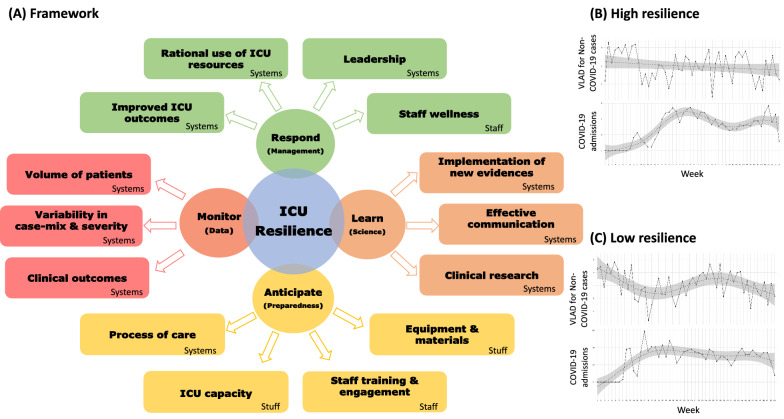
Resilience in Intensive Care Units. In **A,** we show the proposed framework of evaluating the resiliency of an ICU, based on the four pillars of Hollnagel’s Resilience Assessment Grid (Respond, Learn, Anticipate, and Monitor) and the 4 S (Staff, stuff, space, systems). A resilient ICU should respond to sudden periods of crisis with adequate management of its resources, including the staff wellness and leadership, to provide improved outcomes. Resilience should be maintained by continuously monitoring ICU data (increased volumes, case-mix changes, and outcomes), the learning process based on science, evidence-based practices, clinical research, and effective communication. Finally, a resilient ICU must be prepared to maintain health services outcomes during surge periods with adequate staff training, management of processes of care and ICU resources, thus reducing the impact on clinical and staff outcomes. We show two examples of ICUs resilience expected behaviors, comparing the dynamics of mortality of non-COVID-19 patients (measured in Variable-Life Adjustment Display—VLAD) and the surge of COVID-19 admissions from January to December 2020: the high resilience ICU **B** shows a steady progression of mortality in non-COVID-19 cases during the pandemic period; whereas in the low resilient ICU **C** the mortality of non-COVID-19 patients shows high variability, with a decrease in VLAD in the COVID-19 surge peak

First, the capacity to adapt to increased case-volume, defined by the total number of cases, occupation rates, transfers, and off-hours discharges. In addition, the increased number of patients presenting high severity (organ failures or severity of illness or decompensated co-morbid conditions) and use of resources (i.e., increased requirement of advanced support). Overall, ICU and in-hospital mortality, ICU LOS, and the rate of ICU-acquired complications should be defined as core measures of resiliency. Others could be added, such as risk-adjusted mortality rates, delayed/denied access to ICU, and process of care measures, such as adherence to evidence-based protocols. A comparative approach could improve the evaluation by measuring the variation of risk-adjusted mortality and LOS. A proposed framework to evaluate the resiliency of an ICU is provided in Fig. 1.

As resilience is not static, using an indicator such as the Variable life-adjusted display (VLAD) could overcome these limitations by reflecting the adaptation and responses using a risk-adjusted metric. The VLAD is often employed to measure healthcare quality and patient outcomes. This tool predicts the likelihood of a patient outcome, and subsequently plots the difference between the predicted and observed outcomes being represented graphically in a sequential (dynamic) way.

In Fig. 1, we describe an average VLAD showing that the ICU outcomes of non-COVID-19 critically ill-patients vary differently when the surge of COVID-19 patients occurs in two distinct resilience scenarios (Fig. 1B, C). We can observe an ICU, where the mortality of non-COVID-19 patients does not change substantially during the surge (Fig. 1B), demonstrating its resilience. In contrast, a low resilience ICU would present a considerable variation (increase) in mortality as the number of COVID-19 patients increases (Fig. 1C). Such evaluation would trigger actions based on the 4S structure and the implementation of evidence-based care practices.

We believe that a resiliency analysis adds a component of preparedness to the usual ICU performance evaluation and outcomes metrics to be used during the crisis and in regular times. In addition, it provides a dynamic perspective through VLAD or variation analysis.

## Conclusions

The COVID-19 pandemic demonstrated the need for a resilient health system. Although this concept has been discussed for health systems, it was not tested in intensive care, where future studies should evaluate this concept to improve ICU organization for standard and pandemic times.

## Data Availability

Not applicable.
